# 2444. Descriptive Analysis and Control Measures of Candida auris Infections: An Experience form a Quaternary Healthcare Center in Saudi Arabia

**DOI:** 10.1093/ofid/ofad500.2063

**Published:** 2023-11-27

**Authors:** Hala A Amer, Sarah Alfaraj, Ziad Memish

**Affiliations:** National Research Center, Cairo, Al Qahirah, Egypt; King Saud Medical City, Riyadh, Ar Riyad, Saudi Arabia; king saud medical city, Riyadh, Ar Riyad, Saudi Arabia

## Abstract

**Background:**

Candida auris is a multidrug-resistant fungal pathogen causing a *worldwide epidemic.* Since C auris has high mortality rate, ambiguous spread, frequent misidentification, and multidrug resistance, therefore it’s considered as a major global public health *threat. In Saudi Arabia (SA), 1^st^ case of* C auris *was identified in 2018.*

**Methods:**

We described patients with C auris positive specimens reported between January 2021 and June 2022 at one of the main healthcare facilities in SA. A multiciliary committee headed by the hospital leadership and involved infection control, infectious diseases, intensive care, nursing, environmental services, pharmacy, and laboratory has been formulated to investigate the potential outbreak among clusters of cases at intensive care unit (ICU). A bundle of containment measures has been applied at ICU including, C auris admission screening, point prevalence testing every two weeks, patients and staff post exposure screening, environmental samples, high level disinfection (fig1), dedicated of patients’ placement and equipment of care , enhancing patient hygiene, universal contact precautions , and frequent education and auding of infection control practices.

**Figure 1:**
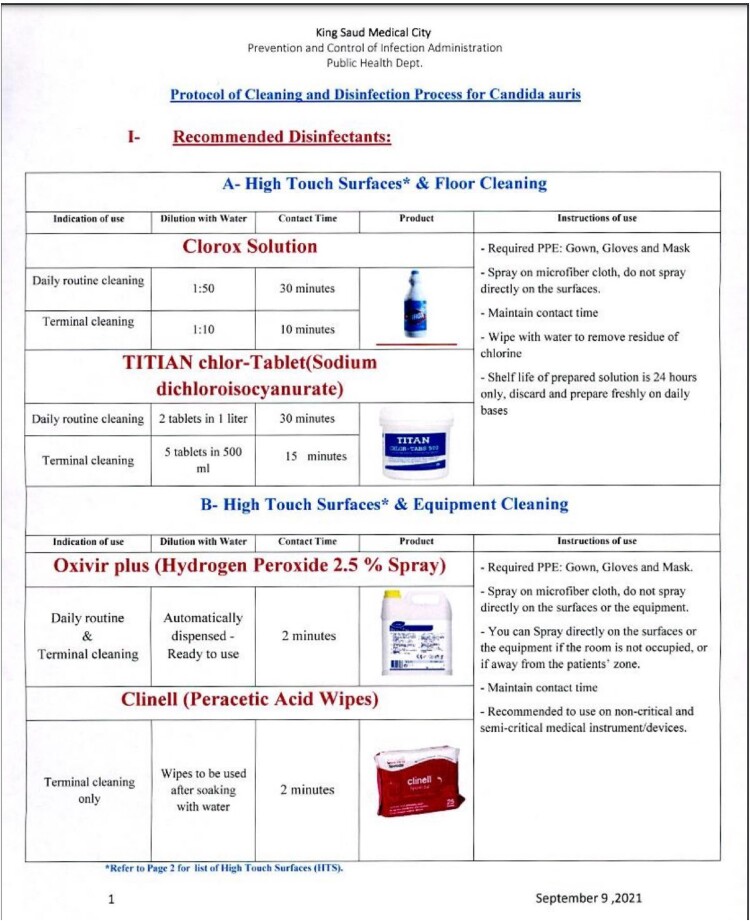
Protocol of Cleaning and Disinfection Process for Candida auris

**Results:**

129 cases of *C auris* were identified in our facility during the study period with two peaks of cases in June 2021 and March 2022. 78.3% were ICU patients with average length of stay 36.2 days. Demographics, comorbidities and risk factors were analyzed (tables1&2). Mortality rate was 43.4% occurred within 20.13 days of infection. Factors significantly associated with mortality were age, trauma patients, ICU admission, vascular access, foley catheters, mechanical ventilation, tracheostomy and endotracheal tube (table 3). Hundreds of exposed staff screened for C auris , none of them turned positive. C auris was isolated from glucometer and body thermometer. The trend gradually decreased following the mitigation actions.

**Figure:**
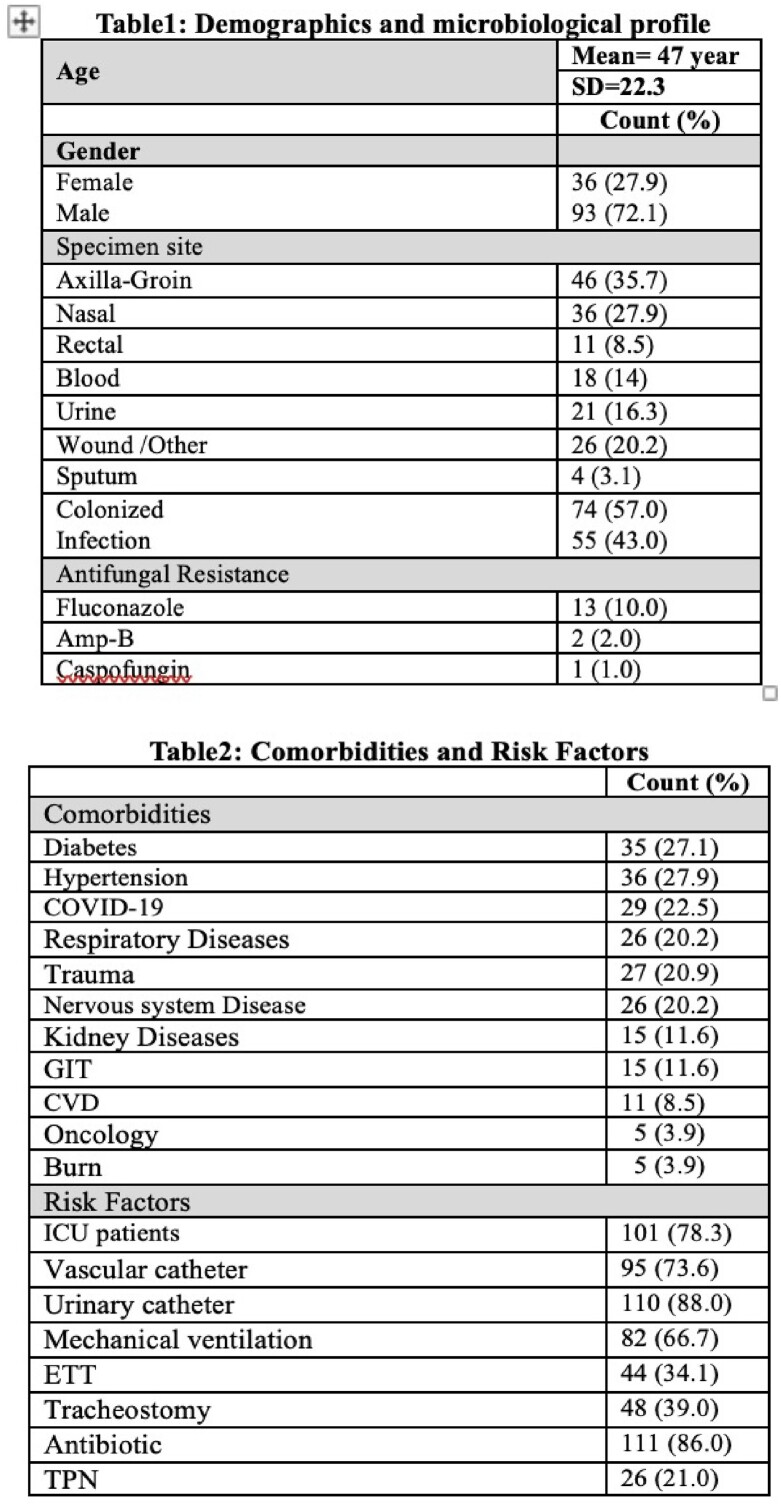

**Figure:**
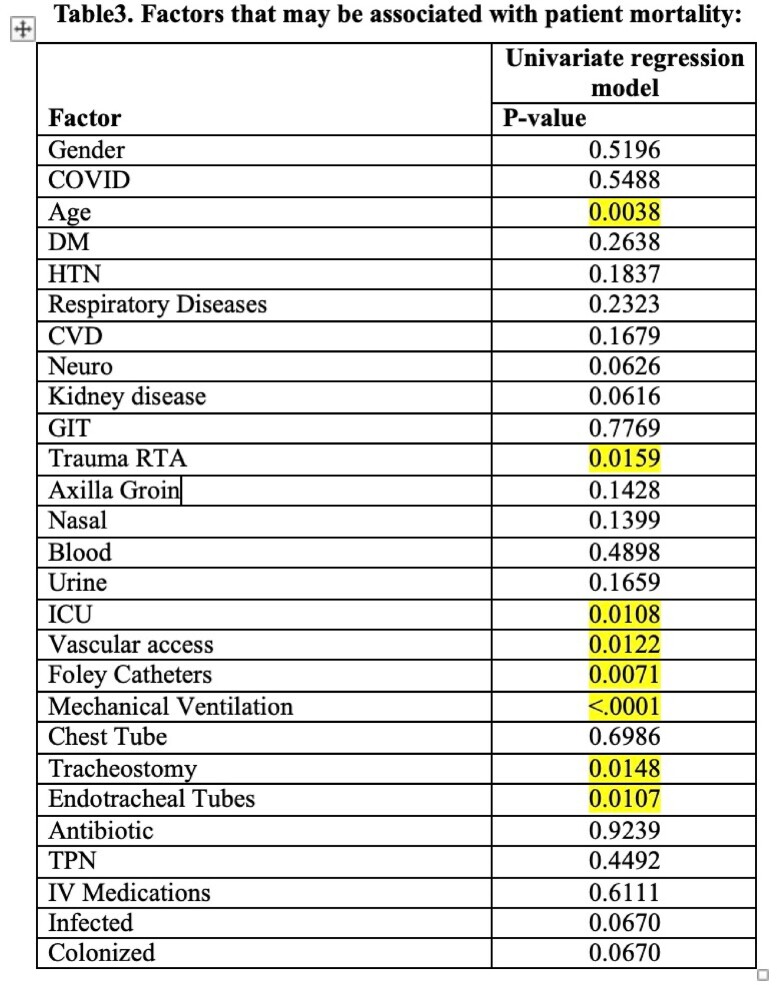

**Conclusion:**

Transmission of *C auris* infection presents a risk to chronic critical patients with multiple medical devices. Early detection through active screening in addition to intensive cleaning of patients care area and equipment, strict adherence to infection prevention, leadership support and team coordination are highly effective to reduce C auris transmission.

**Disclosures:**

**All Authors**: No reported disclosures

